# Rising prevalence of BMI ≥40 kg/m^2^: A high‐demand epidemic needing better documentation

**DOI:** 10.1111/obr.12986

**Published:** 2020-02-04

**Authors:** Kath Williamson, Amy Nimegeer, Michael Lean

**Affiliations:** ^1^ School of Medicine, Dentistry and Nursing University of Glasgow Glasgow UK; ^2^ MRC/CSO Social and Public Health Sciences Unit University of Glasgow Glasgow UK; ^3^ NHS Lothian Edinburgh UK

**Keywords:** BMI ≥40, data quality, prevalence, severe obesity

## Abstract

Whilst previously rare, some surveys indicate substantial increases in the population with body mass index (BMI) ≥40 kg/m^2^ since the 1980s. Clinicians report emerging care challenges for this population, often with high resource demands. Accurate prevalence data, gathered using reliable methods, are needed to inform health care practice, planning, and research. We searched digitally for English language sources with measured prevalence data on adult BMI ≥40 collected since 2010. The search strategy included sources identified from recent work by NCD‐RisC (2017), grey sources, a literature search to find current sources, and digital snowball searching. Eighteen countries, across five continents, reported BMI ≥40 prevalence data in surveys since 2010: 12% of eligible national surveys examined. Prevalence of BMI ≥40 ranged from 1.3% (Spain) to 7.7% (USA) for all adults, 0.7% (Serbia) to 5.6% (USA) for men, and 1.8% (Poland) to 9.7% (USA) for women. Limited trend data covering recent decades support significant growth of BMI ≥40 population. Methodological limitations include small samples and data collection methods likely to exclude people with very high BMIs. BMI ≥40 data are not routinely reported in international surveys. Lack of data impairs surveillance of population trends, understanding of causation, and societal provision for individuals living with higher weights.

## 1 INTRODUCTION

Prior to the 1970s, body mass index (BMI, kg/m^2^) ≥40 was rare.^1^ It was regarded as a pathological condition, deemed to indicate genetic, endocrine, or other vulnerability, affecting a tiny, fixed population, with numbers too small for confident analysis.^2^ Whilst prevalence can be minimal in low‐ to middle‐income countries, contemporary studies suggest rising prevalence, with a current estimated global prevalence of 0.64% in men and 1.6% in women.^3^ It is predicted that by 2025, the global numbers of underweight women will be surpassed by those with BMI ≥35.^3^

Although numbers may appear relatively small, compared with BMI ≥30, frontline health professionals report increasing challenges in providing safe and effective overall health care for people with very high BMIs.^4^ Emerging issues relate to providing for basic care needs such as appropriate positioning and handling,^5^, ^6^ continence and skincare,^7^ but also cover medical problems such as appropriate dosage of medicines,^8^ difficulty in performing medical imaging^9^ and complex psychological issues, including stigma, which impact treatment adherence.^10^ Evidence from several countries indicates that professional guidance or training about severe obesity for care givers is minimal,^11^, ^12^, ^13^, ^14^, ^15^ threatening quality of care for this population,^16^ largely due to lack of awareness and limited evidence base. This problem is compounded by stigmatization over size and weight by the media, within society and among health professionals.^17^

Furthermore, conventional behavioural weight management interventions have minimal impact for BMI ≥40.^18^ Only a minority of individuals access bariatric surgery,^19^ and effective nonsurgical interventions are not yet widely available.^20^ Thus, once individuals reach a very high BMI, the potential for sustained weight loss to improve quality of life and reduce secondary medical complications is limited. The severity and impact of muscular‐skeletal complications reduce physical activity and mobility, increasing dependence on others and putting weight loss further out of reach. Consequently, individuals with BMI ≥40 face reduced life expectancy,^21^ multimorbidity,^22^ disability,^23^ and reduced quality of life.^24^ In turn, this disease burden produces multifaceted demands on health and social care services, raising direct and indirect costs.^25^ Until recently, evidence on direct costs of BMI ≥40 have been limited.^25^ Total health care costs in the United Kingdom rise linearly, and double, as BMI increases^26^ from 20 to 40. A recent systematic review of international health care costs and BMI found costs for people with BMI ≥40 to be 50% greater than for people with BMI 18.5 to 24.9.^27^ Costing studies typically exclude underresearched wider care costs, such as social care^28^ and nursing home usage related to functional disability and often long‐term provision,^28^, ^29^ so current estimates are likely to underestimate the full costs. Forecasts indicate that increased resource usage will continue, including costs required to structurally adapt care facilities to the needs of people with BMI ≥40, alongside providing suitable equipment and training for staff, that is currently missing.^30^

The present scoping review explores the extent of international prevalence data on BMI ≥40. It focusses on measured data, given the potential for error and bias with self‐reported anthropometry by individuals who are overweight.^31^, ^32^ We assessed the extent and quality of epidemiological reporting for the BMI ≥40 category internationally, with a view to improving the documentation of this emerging high‐demand population in future national surveys, to enable development of a reliable evidence base to guide effective care.

## 2 METHODS

The primary epidemiological reports being investigated are health surveys, undertaken by governments for population surveillance to inform strategic policy priorities. Health surveys are not usually reported in the academic literature, unless for a secondary analysis focussed on a specific issue or subpopulation, often with considerable time lag. Thus, database search terms for the primary survey would need to be very broad, making identification through academic databases highly resource intensive, with results prone to being incomplete and outdated. For the present scoping review, an alternative search strategy was therefore applied, based on the sources identified by the most recent systematic review of international BMI survey data, with additional searches to update and supplement these sources, outlined below.

Four key approaches were used to identify potential data sources:

1. Building on previous work

The NCD Risk Factor Collaboration (NCD‐RisC) 2017 study on global BMI trends was chosen as a basis for the initial search, on the basis of its size and rigour: It used 2416 sources of measured data, collected up to 2016.^33^ These sources were compiled from a systematic medical database search, supplemented by a worldwide network of researchers identifying and accessing national measurement surveys via interested parties, including World Health Organization (WHO). Full details are given in the published paper and its appendix.^33^ All 2416 sources in the NCD‐RisC appendix were screened as per the inclusion criteria in Table 1, with digital snowball searching used to locate individual data sources.

- Digital searching of current grey literature sites

**Table 1:** Source inclusion and exclusion criteria

Included	Excluded
Reports BMI ≥40 prevalence	Does not report BMI ≥40 prevalence
Measured anthropometric data	Self‐reported anthropometric data
Nationally representative data: sampling strategy showing national coverage based on electoral roll/census or similar, with at least stratification for age and sex	Nonnationally representative, subpopulations by age/sex/rural/urban/regional/community
Adults aged 15 years or older	Children and adolescents
Data collected in or since 2010	Data collected pre‐2010
Data already compiled in a publicly available report/website^a^	Data requiring registration/searching through raw data^b^
Report in English language	Report not in English language
Not already identified by search	Already identified through alternative source

Abbreviation: BMI, body mass index.

a For immediate use by decision makers.

May be available to academics, but requiring analysis and presentation prior to use by decision makers.

Searching of key international organizations websites known to compile BMI population survey data was undertaken, focussing on the Organization for Economic Cooperation Development (OECD), the WHO, and the World Obesity Federation (WOF).

- Systematic database search

To ensure identification of any new sources since 2016 in the academic literature, a modified version of the NCD‐RisC literature search was undertaken in Medline and Embase using the search terms in Appendix A. Results from conference proceedings were deliberately included, as they can provide the first exposure of research analysis, highlighting new or updated data sources, whilst full articles commonly take much longer to publication, if indeed they are ever published in full. Sources were searched both for BMI ≥40 data, but also to identify any new sources/health surveys not already located through parts 1 and 2 of the search process.

- Digital snowball searching

Whilst NCD‐RisC identified sources, individual sources needed located and accessed, often directly from a website of government agencies or international bodies. This was undertaken digitally, seeking previously unknown sources or articles.

**Figure 1:**
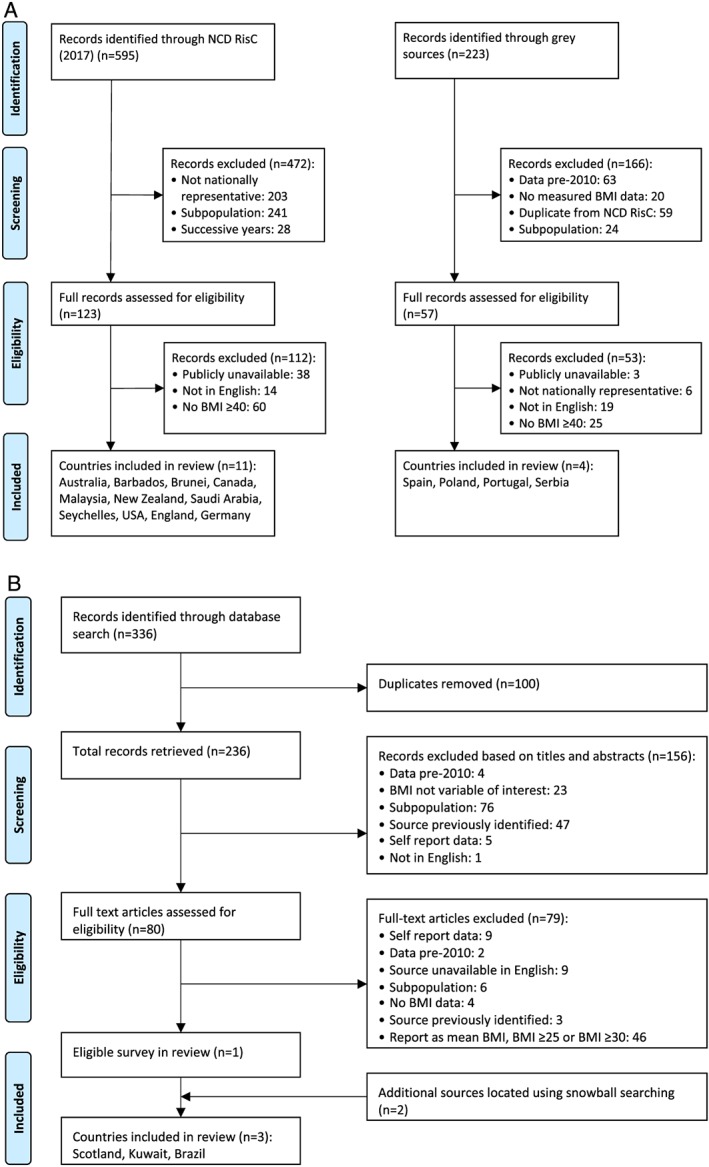
Search strategy (A) part 1 and 2: NCD‐RisC sources and grey sources, (B) part 3 & 4: literature search

All sources were screened using inclusion criteria in Table 1. Figures 1A and 1B represent the search process, with numbers of studies included and excluded at each stage. Searches took place between June and September 2019. Where successive or annualized surveys were identified, sources were checked for the most current data, with the most recent data retained. If multiple studies for the same country or source were identified, the one with the largest sample, or that provided the most information to data extraction, was retained. These pragmatic methods adopt a systematic approach to exploring the broad evidence landscape in an emerging area, highlighting gaps for future, more detailed research.^34^

## 3 RESULTS

Prevalence data of measured BMI ≥40 from 2010 onwards were located for 18 individual countries, on all five continents, comprising 12% of eligible national survey data examined (Table 2) either in its primary form (n = 10) or through secondary analysis (n = 8). Data were very recent, with half of the countries reporting prevalence from 2015 onwards, and only three back to 2010. None of the original sources located reported BMI data categories higher than BMI ≥40 (for example, BMI ≥45/≥50), with just one secondary analysis from Spain doing so.^41^

**Table 2:** International BMI ≥40 kg/m^2^ prevalence rates for data collected since 2010

Country	Prevalence %			Source	Year Data Collected	Sample Size	Age, years	Ongoing Data Collection	Data Collection Method	Scale Capacity, kg
	All	Men	Women							
WHO European Region										
England	4^a^ (3.1‐4.1)	2^a^ (1.9‐3.2)	5^a^ (3.9‐5.4)	Health Survey England^35^	2017	6530	≥16	Annual	Home visit	200
Scotland	3^a^ (2.8‐4.1)	2^a^ (1.5‐3.2)	4^a^ (3.5‐4.5)	Scottish Health Survey^36^	2018	3746	≥16	Annual	Home visit	200^b^
Germany	—	1.2 (0.8‐1.8)	2.8 (2.2‐3.5)	German Health Interview and Examination Survey for Adults^37^	2008‐2011	7116	18‐79	No	Community centres^c^	250
Poland	—	1.3 (0.09‐1.7)	1.8 (1.3‐2.3)	WOBASZ II^38^	2013‐2014	5417	≥20	No	Home visit and clinics	No data
Portugal	1.8 (1.4‐2.2)	—	—	National Health Examination Survey (INSEF)^39^	2015	4819	25‐74	No	Health centres	200
Serbia	1.4 (1.2‐1.6)	0.7 (0.5‐0.9)	2.0 (1.7‐2.4)	National Health Survey^40^	2013	13 103	≥15	No	Home visit	No data
Spain	1.3 (1.0‐1.8)	1.0 (0.6‐1.6)	1.6 (1.1‐2.3)	Nutritional Study of Spanish Population (ENPE)^41^	2014‐2015	3966	25‐64	No	Home visit	150
WHO Eastern Mediterranean Region										
Kuwait	5.5 (4.8‐6.3)	3.9 (2.9‐4.9)^d^	7.0 (5.9‐8.1)^d^	STEPS, Weiderpass et al^42^	2014	3589	18‐69	No	Health centres	No data
Saudi Arabia	—	2.5 (2.3‐3.3)	4.7 (3.9‐5.5)	Saudi Health Interview Survey^43^	2013	10 337	≥15	No	Home visit	No data
WHO Western Pacific Region										
Australia	4.0 (3.6‐4.4)	3.3 (2.8‐3.8)	4.7 (4.2‐5.2)	National Health Survey^44^	2017‐2018	18 656	≥18	3‐4 yearly	Home visit	200
New Zealand	5.1 (4.5‐5.6)	3.5 (2.9‐4.1)	6.6 (5.8‐7.4)	New Zealand Health Survey^45^	2017‐2018	13 869	≥15	Biennial	Home visit	200
WHO Africa Region										
Seychelles	–	1.5 (0.5‐2.5)	6.7 (4.9‐8.5)	Seychelles Heart Study IV^46^	2013‐2014	1240	25‐64	No	Health centre	No data
WHO Americas Region										
Barbados	5.0 (3.7‐6.7)	1.8 (0.8‐4.0)	7.9 (6.0‐10.5)	Barbados Health of the Nation Study^47^	2011‐2013	1197	≥25	No	Home visit	No data
Brazil	1.8 (1.6‐2.0)	—	—	National Health Survey, Wagner et al^48^	2013	49 359	20‐64	No	Not given	No data
United States	7.7 (6.6‐8.9)	5.6 (4.3‐7.2)	9.7 (8.4‐11.2)	NHANES, Hales et al^49^	2015‐2016	5337	≥20	Biennial	Mobile Examination Centre	272
Canada	4.0 (2.8‐5.8)	2.6^e^ (1.3‐5.0)	5.5 (3.9‐7.7)	Canadian Health Measures Survey^50^	2014‐2015	5794	18‐79	Biennial	Mobile Examination Centre^c^	272^d^
WHO South East Asia Region										
Brunei Darussalam	3.0 (2.2‐4.0)	3.9 (2.6‐5.6)	2.3 (1.4‐3.6)	National Health and Nutrition Status Survey^51^	2010‐2011	1524	≥19	No	Local clinic^c^	200
Malaysia	1.4 (1.2‐1.6)	0.9 (0.7‐1.2)	1.9 (1.6‐2.3)	National Health and Morbidity Survey^52^	2015	5196	>18	No	Not given	150

Abbreviation: BMI, body mass index; WHO, World Health Organization.

Reported as rounded, whole numbers only.

Confirmed through personal communication with survey team.

Transport provided to centre if needed.

Confidence intervals calculated using % sample size for men and women, as absolute numbers only provided for all adults.

Source advises use with caution.

Germany,^37^ Saudi Arabia,^43^ Seychelles,^46^ and Poland^38^ report only for men and women separately, with no data for combined‐sex adults. In contrast, only combined‐sex‐adult data were available for Brazil^48^ and Portugal.^39^ All regions of the world (as defined by WHO) contained a country with rates above 4% for women, and 2% for men, with the exception of Africa, where the maximum was Seychelles with 1.5% for men.^46^ Other than Brunei Darussalam,^51^ rates for women were universally higher than for men.

**Figure 2:**
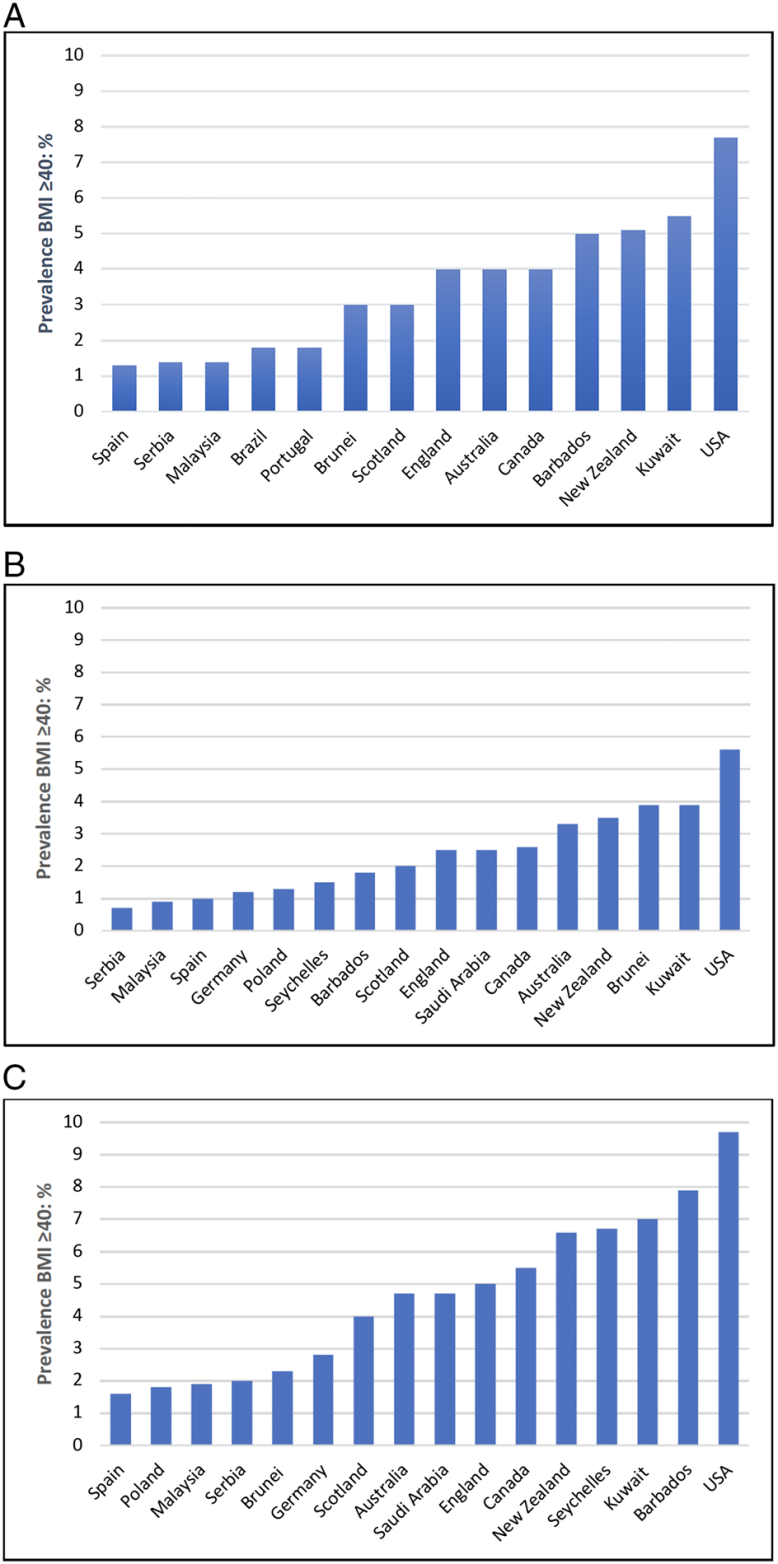
International body mass index (BMI) ≥40 prevalence rates by country: (A) all adults, (B) men, and (C) women

Prevalence rates are presented graphically in Figure 2A‐C. The United States has the highest prevalence,^49^ with rates across all adults, men, and women ranging between 5.6% and 9.7%. Other countries with higher rates (5.0%‐7.7%) for all adults and women are New Zealand,^45^ Kuwait,^42^ and Barbados,^47^ although rates in men are markedly lower. Australia,^44^ Canada,^50^ Scotland,^36^ England,^35^ and Saudi Arabia all have rates in the region of 2.5% to 5.5% for all adults and/or women, again with men notably lower. Germany, Serbia,^40^ Spain, Portugal, Poland, Brazil, and Malaysia^52^ display the lowest rates between 0.7% and 2.8%. Brunei Darussalam and Seychelles both exhibit disparate prevalence patterns from those above for men and women.

Table 2 is modelled on Foresight's International Evidence Review,^53^ which when published illustrated the general availability of regionally comparative BMI ≥30 prevalence data. The rigorous inclusion criteria applied during searching means that most sources (with potential exception of Spain and Poland) were from surveys done or supported by national government agencies, with primary reporting through grey literature sources. Due to the constraints associated with gathering measured data, total sample sizes tend to be relatively small, with 13 countries being <8000, and five above this. With prevalence under 10% of these numbers, wide confidence intervals result for BMI ≥40, as seen in Table 2.

Less than half of the countries included have regular surveillance, ranging from annual to three to four yearly (Table 2: England, Scotland, Canada, New Zealand, Australia, and the United States), significantly limiting analysis of trends over time. Reporting from other countries appears to be more ad hoc and unpredictable, without planning for regular surveillance.

### 3.1 International survey data sources

Globally, BMI ≥40 data were generally poorly available compared with BMI ≥30 data. Seventy‐one NCD‐RisC sources had BMI ≥25/≥30 data available in English, of which only 11 (15%) provided BMI ≥40 data. Forty six studies out of the 80 screened for the literature search reported mean BMI or BMI ≥25/≥30 data, with just one (2%) reporting BMI ≥40. For Africa, only the island state of Seychelles presented BMI ≥40 data.

The OECD annually publishes country by country Health Statistics with measured data sources for 26 out of 44 countries in its 2019 report.^54^ Many major European countries are missing, as the European Health Interview Survey (EHIS), which is the primary health data collection tool for Eurostat, the Statistics office of the European Union, uses self‐reported height and weight data.^55^ The European Health Examination Survey does include measured data but appears not to have been widely adopted.^56^

Additional grey sources were searched, including WHO website, where the categorizations consistently applied to data were BMI 25.0 to 29.9 and ≥30, with no additional categorization for BMI ≥40. The WOF website supports the Global Obesity Observatory, featuring a searchable interactive map detailing national overweight and obesity rates, with data sources referenced. All 179 available countries were individually searched, with 12 displaying data on BMI ≥40,^57^ of which four had not been previously identified and fitted the inclusion criteria.

### 3.2 Data quality

To enable assessment of data quality, the column headers of Table 2 highlight basic quality parameters appropriate to national health surveillance,^58^ with additional categories particularly relevant to the population with BMI ≥40.

Survey methods for the six countries with regular surveillance in place (England, Scotland, Australia, New Zealand, Canada, and the United States) are available for scrutiny on public websites. These surveys employ complex sample design, aimed at reducing bias and with weighting to reflect the age/sex stratification of the population. Response rates are difficult to compare, due to heterogeneity of definitions. As ongoing programmes, the surveys measure nonresponse across years, which appears to be a growing challenge, with Australian data showing the nonresponse rate for the BMI module specifically, increasing from 26.8% in 2014 to 2015 to 33.8% in 2017 to 2018.^59^ Secondary analyses tended to have less available detail on sampling and methodology, with those for Spain^41^ and Brazil^48^ referencing previous publications not available in English.

Data collection methods of included surveys were scrutinized for factors that may affect participation of people with BMI ≥40 (Table 2). Eleven countries specifically stated exclusion criteria, which were very similar, namely, the institutionalized population. This excluded those in hospitals and, apart from New Zealand, people in care homes. No surveys appeared to document an upper BMI limit, but the capacity of scales used has potential to enact this, effectively meaning individuals with weights over the maximum capacity of the instrument are either excluded or estimated self‐reports.^35^ No clear data on scale capacity could be found for seven countries, two countries appear to have used scales with a maximum capacity of 150 kg, whilst nine had scale capacities of 200 kg or over. Functional mobility limitations may affect participants' ability to stand on scales or attend examination centres outside the home. For two countries, place of anthropometry data collection was unavailable, nine countries used home visits, whilst seven required participants to leave their home, although some of these offered transport if needed.

## 4 DISCUSSION

Whilst good quality BMI ≥30 prevalence data are now available globally, largely due to the widespread implementation of surveillance tools such as STEPS, there are few robust measured data on BMI ≥40 or higher categories. The published surveys generally use similar proven methodologies to obtain population‐representative data. However, the methods used to assess BMI ≥30, risk providing inaccurate results for higher BMI categories, for example, if they require mobility of participants or if the scales used have an upper limit of 200 kg. Hence, true prevalence may be higher than stated in Table 2.

**Figure 3:**
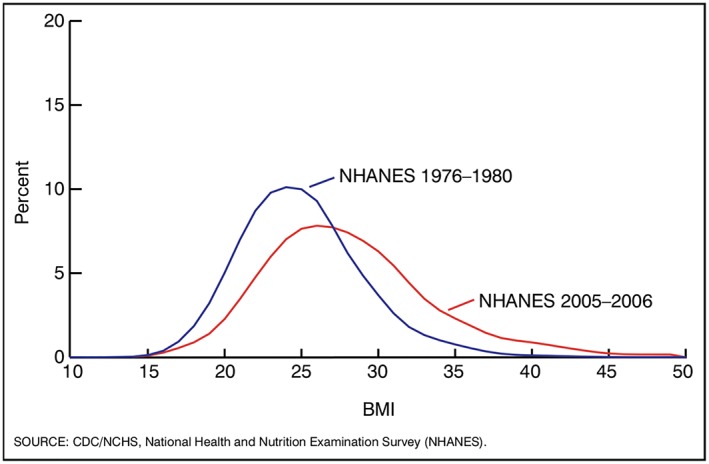
Changes in the distribution of body mass index (BMI) between 1976‐1980 and 2005‐2006, adults aged 20 to 74 years: United States.^63^ Data are age adjusted by the direct method to the year US Census 2000 estimates using age groups 20 to 39, 40 to 59, and 60 to 74. Overweight is BMI of 25.0 to 29.9 kg/m^2^; obesity is BMI at or above 30.0 kg/m^2^; and severe obesity is BMI at or above 40.0 kg/m^2^. Pregnant women are excluded from the analysis. Sources: NCHS, National Health Examination Survey, and National Health and Nutrition Examination Surveys

The data that are available have limitations, notably wide confidence intervals for higher BMI categories, making it difficult to determine the significance of annual changes. If, as predicted, numbers with BMI ≥40 continue to grow, this problem may diminish, although is unlikely to disappear altogether as the cost of gathering measured data limits sample sizes. The limited long‐term trend data available help to overcome uncertainties with large confidence intervals for the smaller numbers with BMI ≥40. Since 1995, BMI ≥40 prevalence in Scotland has trebled for women aged 16 to 64 years.^60^ Australia saw similar increases of 2.9‐fold for men and 2.0‐fold for women between 1995 and 2011 to 2012.^61^ Prevalence of BMI ≥40 in the United States quadrupled between 1976 and 2004, substantially surpassing the rise in BMI ≥30.^62^ Prevalence in men in England aged 16 years or older experienced an eight‐fold increase since 1995,^35^ but Brazil reported the most dramatic increase of nearly 20‐fold between 1974‐1975 and 2013.^48^ Overall population distributions have thus consistently shifted upwards, as illustrated in Figure 3, comparing waves of US NHANES data from 1967‐1980 to 2005‐2006.^63^

Comparison with analyses of self‐report surveys from the United States and Canada, which allow for much larger samples, all show disproportionately larger growth in higher BMI categories, with increases of up to 10‐fold in the BMI ≥40 category.^64^, ^65^, ^66^ This is despite suggestions that the underestimation of weight for self‐report data is likely to be greater with higher BMI.^31^ Thus, despite wide confidence intervals in individual survey years and some variations in methodologies, measured trend data evidence from several countries, together with larger‐scale self‐report datasets, all support a long‐term rise in BMI ≥40.

Two key sources of data for NCD‐RisC and WOF were STEPS reports and Demographic Health Survey (DHS) country reports. The STEPwise approach, featuring three different level of “steps” of key risk factor assessments for NCDs, was developed by WHO, to aid countries increase their surveillance capacity.^67^ One hundred and thirteen country reports or data sheets were publicly available on the WHO STEPS site at the time of searching.^68^ DHS is supported by the US Agency for International Development (USAID), to inform planning particularly in relation to maternal and child health, with over 93 standard DHS reports since 2010 on its website.^69^ Both of these are easily accessible population surveillance tools, widely used by low‐ to middle‐income countries, with standard methodologies including collecting measured height and weight in a household setting. Current standard reporting practice for STEPS and DHS focusses on BMI 25.0 to 29.9 and ≥30, with no additional categorization for BMI ≥40.

International health surveillance provides reliable health information that is comparable over time and between populations. It has allowed documentation of a nutrition transition that is rooted in the impact of large‐scale social changes such as reduction in physical activity, increased urbanization, and greater consumption of processed food.^70^ Whilst regional variation remains, the major global concern has moved away from underweight as the primary nutrition issue, towards excess weight as the leading cause of morbidity and mortality through secondary noncommunicable diseases.^23^ This has already occurred to such a degree that BMI ≥35 in women now surpasses underweight in 165 countries for women and 113 for men.^3^ Yet current national documentation of BMI distribution does not reflect this shift, with the focus still on reporting categories <18.5 to ≥30, and/or mean BMI, as the DHS and STEPS reports evidence. This historical bias is obscuring the significant changes in higher BMI categories.^42^, ^66^ Without characterizing this progression, policymakers and planners are unable to respond effectively to the needs of the population or evaluate the effectiveness of policies.

The example of Kuwait in this review provides a case in point. The original 2014 STEPS report on the WHO website documents the mean BMI and four separate BMI categories <18.5, 18.5 to 24.9, 25.0 to 29.9, and ≥30, without ≥40, as a distinct category.^68^ The BMI ≥40 data were later reported by the analysis of Weiderpass et al of the original STEPS dataset, illustrating that the data had been collected but gone unreported.^42^ Table 2 shows Kuwait's prevalence rates as second highest only to the United States, providing valuable information regarding BMI population distribution, in a region where no other sources of all adult prevalence were found.

One practical solution would be for WHO and similar agencies to call for data on the BMI ≥40 population to be included when reporting all anthropometry surveys. The National Child Measurement Programme in England took an equivalent step in 2018, adding severe obesity as a reporting category.^71^ Given that the prevalence of BMI ≥50 is now similar to that of the BMI ≥40 category about 20 years ago, with some real‐world datasets including categories of 50/60/70,^72^ it may be wise also to include reporting BMI ≥50, to map future trends. This would hugely increase the amount of BMI ≥40 data available globally, with little extra cost, given that the data are already collected. For countries with small numbers to report, the need for caution in interpretation would be dealt with in the same way for low numbers in any category, for example, underweight.

### 4.1 Causation

The lack of data on BMI ≥40 trajectories by region, nation, age, sex, and class makes it difficult to explore causation. Improved international data, ideally from longitudinal studies, would promote comparison between countries, taking into account their differing social and economic contexts.^73^ Together with the emergence of large‐scale genome studies looking at the inherited susceptibility to BMI ≥40,^74^ reasons for the escalation of high body weight may be more accurately sought. There are some indications of associations with lower socio‐economic status in some populations,^75^, ^76^ but these patterns require further study. Concerningly, some countries report increasing rates of the highest BMI groups growing for children and adolescents,^71^, ^77^ with potential for excess weight to track through into adulthood. This would differ from current patterns, when rates are lower in early adulthood, peaking in middle age.^35^, ^49^, ^78^ Unusually, the survey data from Brunei Darussalam showed 19‐ to 29‐year‐olds having some of the highest rates of BMI ≥40 across the age trajectory.^51^

A lack of prevalence data keeps the population hidden, preventing development of appropriate weight management services to treat this population group, along with comparative analysis of different treatment models and health care systems.^25^ BMI ≥35 with comorbidities or BMI ≥40 with or without comorbidities is a commonly applied threshold for bariatric surgery, yet access to surgery is often very limited.^19^, ^79^ Evidence on effective alternatives to surgery or prevention is needed, whilst access to traditional services can be difficult for people with BMI ≥40 due to functional disability.^80^ Improved global prevalence data would facilitate work on economic costing of treatment and prevention for the population with BMI ≥40.

### 4.2 Consequences of rise in prevalence

Whilst numbers may appear small in terms of proportion of the whole population, given that these are at national scale, they translate into significant absolute numbers with a large real‐life impact on care provision.

#### 4.2.1 Health risk and comorbidities

It is well recognized that BMI can be a poor proxy for body fat, and in many studies, waist circumference or evidence‐based predictive equations for total body fat are better than BMI for assessing health risk, particularly cardiac and metabolic health outcomes.^81^, ^82^, ^83^, ^84^ Consequently, surveys from the United States, England, Scotland, New Zealand, and Australia all measure waist circumference. However, people with BMI ≥35 exceed these cut points, often with a large abdominal fat apron, making methods which incorporate waist circumference unlikely to be reliable for very high body weights. Thus, BMI remains the best available simple estimate of body fat at the highest levels. Surveys and studies need to consider data collection methods, particularly scales capable of weighing at least 200 kg, with easily accessible wide and low platforms, ideally offering home visits to facilitate accurate data collection from people with high BMIs. Additionally, a review is needed regarding the treatment of what have historically been seen as biologically implausible values (BIVs), the majority of which have been found to be accurate.^85^ The definition of BIVs and use of upper thresholds for weight and BMI in research studies requires reexamination in view of the documented shift in population distribution towards heavier BMIs.

Elevated health risk translates into increased prevalence of multimorbidity (co‐occurrence of ≥2 conditions) compared with those of normal weight by nearly two^86^ to seven times,^40^ particularly for cardiometabolic multimorbidity.^22^ Alongside physical diseases, risk of depression increases with BMI,^87^ together with functional disability,^88^ with increasing numbers of people with BMI ≥40 living in care homes.^29^ As such care of these multiple obesity‐related consequences amplifies health care costs considerably.^89^ Given the rise in prevalence and size of these costs, in addition to older costing studies being limited by BMI thresholds that are now too low, this is likely to become of increasing concern globally.

#### 4.2.2 Planning

As an emerging population, people with BMI ≥40, especially those with BMI ≥50 and ≥60, have needs that are currently often unaddressed by service providers.^4^ Increasingly, changes to care environments are being needed to accommodate larger body sizes, requiring adjustments such as widened doorways, reinforced floors, suitable seats, and larger rooms. Problems featured in the health literature demonstrate the scope of challenges, such as evacuation planning,^90^ diagnostic scanning,^9^ and positioning during surgery in theatre.^91^ Documented requirements include staff training,^11^ specialist equipment provision,^91^ and development of specialist clinical protocols covering essential areas, such as tissue viability guidance.^92^ However, issues are not restricted to health care but affect all aspects of life, including specialist housing,^93^ adapted workplace design,^94^ larger sized fashionable and safety clothing,^95^ and barriers to travelling.^96^ Projections of future severe obesity prevalence using different datasets agree in predicting continued rises for the foreseeable future.^1^ Current analyses suggest five million living with a BMI ≥40 in the United Kingdom by 2035, with rates up to 20% for Welsh women aged 55 to 64 years^97^ and over 20 million people affected in the United States.^30^ Better characterization of the population with BMI ≥40 is required to support organizational and societal readiness for this population. Adaptations are expensive in time and money, particularly when made retrospectively, underlining the need for accurate planning to happen now.

### 4.3 Strengths and limitations

This review has concentrated on robustly measured data to establish the international prevalence rates for BMI ≥40. Whilst the rationale for exclusion of self‐report data is sound, in that it commonly underestimates BMI, the exclusion also acts as a limitation, for example, by excluding EHIS data, which covers many European countries. The sample sizes possible with measured data are reduced by the need for resources to make measurements, and there is potential bias against including very heavy individuals whose mobility is impaired. In some cases, the upper limit of scales excluded the heaviest individuals. These limitations would tend to underestimate the true prevalence of the highest BMI categories, not overestimate.

Applying a lower threshold of BMI ≥35 would have broadened the available data, whilst potentially weakening the focus on the highest BMI category, where costs and clinical complexity is greatest. Some studies report the lower threshold of BMI ≥35 particularly those examining Asian populations where different BMI cut‐offs relating to overweight and obesity are often applied.^98^

Limiting the review to the English language prevented examination of some original data sources, which could only be located in their native language, for example, Spanish for Chile and Mexico. It was not possible to locate English versions of these, and resources did not allow for translation. The OECD reports these original sources in English in its database, but only at BMI thresholds of 25.0 to 29.9 and BMI ≥30, with prevalence for Chile and Mexico the highest in the world, above even those of the United States.^99^ Thus, they are likely to have significant BMI ≥40 prevalence. Additionally, whilst the search processes were broad, encompassing a variety of sources and used systematic methods, they were not exhaustive, as might be expected from a formal systematic literature review or meta‐analysis. We believe that they represent a reliable summary of the current evidence base on BMI ≥40, as it is available to decision makers. Some individual sources have not been included, notably those from non‐English publications, but they are unlikely to alter the very consistent conclusions.

## 5 CONCLUSION

This review highlights the poor availability of robust international data available on the emerging issue of BMI ≥40 prevalence. The measured data available suggest significant prevalence on all five continents, with proportionally large rises in recent decades. Given the multiple care challenges, high resource needs and poor current evidence base for this population, routinely reporting BMI ≥40 and higher categories in national surveys, would be valuable, with appropriate caveats for interpreting the still small numbers in individual surveys. Accurate characterization of the subpopulations with BMI ≥40 and higher categories requires consideration of measurement equipment and the mobility limitations of individuals with high BMI.

## CONFLICT OF INTEREST

The authors declare no competing financial interests in relation to this work.

## FUNDING INFORMATION

Kath Williamson was supported by an NHS Research Scotland Career Researcher Fellowship, an RCN Professional Foundation Bursary, and an NHS Lothian Research Futures award for this work.

Amy Nimegeer is funded by the Medical Research Council (MRC) and the Chief Scientist Office of the Scottish Government Health Directorates (CSO) as part of the MRC/CSO Social and Public Health Sciences Unit's Informing Healthy Public Policy programme (MC_UU_12017/15 and SPHSU15).

Michael Lean is funded by the University of Glasgow.

## APPENDIX A

Search strategy and terms, last search undertaken on 28 September 2019.

Medline (Ovid) and Embase

(National health survey or national population surveillance or national prevalence or epidemiological survey).tw

(BMI or body mass index or obes*).tw

Limited to humans, (all adults (19 plus years)), English language, 2016 – current, All types of publication

(Child or children or adolescents or baby). Tw records remove
